# Integrated endoscopic thoracolumbar mini‐hemilaminectomy and lateral corpectomy in cadaver dogs

**DOI:** 10.1111/vsu.14316

**Published:** 2025-07-18

**Authors:** Megan M. Wolfe, Lisa R. Bartner, Eric Monnet, Katherine Neal

**Affiliations:** ^1^ Department of Clinical Sciences Colorado State University Fort Collins Colorado USA

## Abstract

**Objective:**

To evaluate the feasibility of minimally invasive integrated endoscopic mini‐hemilaminectomy and thoracolumbar lateral corpectomy at six intervertebral disc spaces in the canine thoracolumbar vertebral column.

**Study design:**

Experimental, randomized cadaveric study.

**Animals:**

Eight cadaver dogs euthanized for reasons unrelated to this study.

**Methods:**

Preoperative computed tomography (CT) scans were performed for surgical planning. Using an endoscopic system, mini‐hemilaminectomy and thoracolumbar lateral corpectomy were performed from the T11–12 through L3–4 intervertebral disc spaces, with cadavers in sternal recumbency or positioned at a 30°–60° angle. Postoperative CT scans were used to measure corpectomy dimensions as a percentage of total vertebral dimensions.

**Results:**

Median ± interquartile range (IQR) corpectomy dimensions were 23.07% ± 16.47% of vertebral body length, 46.76% ± 21.7% of vertebral body height, and 74.44% ± 47.37% of vertebral canal diameter. Targeted dimensions were achieved at all surgical sites from T11–12 through L3–4.

**Conclusion:**

Integrated endoscopic thoracolumbar lateral corpectomy was successfully performed at all intervertebral disc spaces from T11–12 through L3–4 using an endoscopic system, although thoracic sites required more practice.

**Clinical significance:**

This pilot study will guide further clinical applications of minimally invasive neurosurgery in small animal patients.

## 1 INTRODUCTION

Minimally invasive surgery is gaining popularity in veterinary medicine. Research in human medicine has shown that minimally invasive techniques allow for a smaller surgical incision, less muscle dissection, preservation of normal anatomical structures, and optimized visualization.^1^, ^2^, ^3^ A recent study evaluating minimally invasive discectomy in dogs found significant reduction in measures of inflammation and postoperative pain compared to conventional hemilaminectomy.^4^ More studies are needed to inform the veterinary neurosurgeon about optimal techniques and how minimally invasive neurosurgery may be used more commonly in clinical practice.

Thoracolumbar lateral corpectomy (TLLC) is a documented technique for decompression of intervertebral disc herniation in dogs, especially in cases of more chronic intervertebral disc protrusions.^5^, ^6^, ^7^, ^8^ Combining a corpectomy with a mini‐hemilaminectomy allows for visualization of the spinal cord and corresponding nerve root during drilling of the corpectomy without significantly increasing vertebral column range of motion.^9^, ^10^ It has been previously documented that minimally invasive mini‐hemilaminectomy and TLLC are feasible via an endoscopic‐assisted technique.^11^ However, minimally invasive mini‐hemilaminectomy and corpectomy has not been evaluated using an entirely endoscopic surgical system, other than one study using a patient‐specific three‐dimensional (3D)‐printed surgical guide.^12^ Additionally, the efficacy of this technique at various intervertebral disc sites in the thoracic and lumbar vertebral column is not documented. Challenges may exist for corpectomy in the thoracic vertebrae compared to the lumbar vertebrae, since the presence of the rib head may hinder adequate exposure of the vertebral body and sometimes necessitates partial rib head resection.^5^, ^6^, ^11^

The primary aim of this study was to assess the feasibility of performing minimally invasive mini‐hemilaminectomy and thoracolumbar lateral corpectomy on cadaver dogs using a currently available integrated endoscopic surgical system. A secondary aim was to assess the dimensions of thoracolumbar lateral corpectomy achieved at each intervertebral disc space from T11–12 through L3–4, and if surgical positioning affected corpectomy slot dimensions using this minimally invasive approach.

## 2 MATERIALS AND METHODS

### 2.1 Experimental design

Eight medium to large‐breed cadaver dogs (range: 15–30 kg) euthanized for reasons unrelated to the present study were obtained from a local animal shelter. The Colorado State University Institutional Animal Care and Use Committee (reference no.: 3976) approved use of cadavers for this study. Cadavers were stored frozen and then thawed for approximately 48 h prior to use for the present study.

Precontrast computed tomography (CT) scan (Siemens SOMATOM Force, 128‐slice; Malvern, Pennsylvania) of the thoracolumbar vertebral column were obtained for all cadavers. Sagittal images extended cranially through the T9 vertebral body and caudally through the sacrum. Transverse images extended from the T9 vertebral body caudally through at least L4, with slices oriented perpendicular to the spinal canal on the sagittal images. Needles inserted into the spinous processes were used as reference for anatomic localization. If any cadaver had evidence of thoracolumbar vertebral pathology such as intervertebral disc herniation or incorrect number of vertebrae, they were excluded from the study.

CT images were used to measure the vertebral body length, height, and width for the T11 through L4 vertebrae. Total vertebral body lengths were measured on midline at the longest aspect from endplate to endplate. Total vertebral body height was measured at the tallest portion of the vertebral body on midline, at the caudal endplate. Total vertebral canal diameter was measured from luminal margin to luminal margin of the vertebral canal at the mid‐aspect of the vertebral body. Using these measurements, target corpectomy dimensions were calculated for each surgical site. Based on previous studies,^5^, ^6^ target corpectomy dimensions were set as 25% of the length of each vertebral body, 50% of the vertebral body height, and 67% of the vertebral body width. Success was defined as meeting or exceeding these target dimensions.

Mini‐hemilaminectomy and corpectomy were performed on each cadaver from the T11–12 through L3–4 intervertebral disc spaces, alternating left and right, with the order of surgeries for each cadaver determined by an online random generator (www.random.org). A standard open thoracolumbar corpectomy can be performed with the patient in sternal recumbency or angled slightly away based on clinician preference.^13^ Surgeries on the first two cadavers at the start of the study (dogs 1 and 2) were performed with the cadavers positioned in sternal recumbency. Surgeries on dogs 3 and 4 were performed with the cadavers positioned roughly 30° away from the surgeon. Surgeries on dogs 5 and 6 were performed with the cadavers positioned at a steeper angle, approximately 60° away from the surgeon, to further explore the effect of angled positioning. Surgeries on dog 7 were performed with the cadaver angled 60° away from the surgeon, and surgeries on dog 8 were performed with the cadaver angled 30° away from the surgeon. Surgeries on dogs 7 and 8 were performed within the same week at the end of the study to reduce the chance of surgeon experience being a confounding factor to the effect of surgical positioning.

**FIGURE 1:**
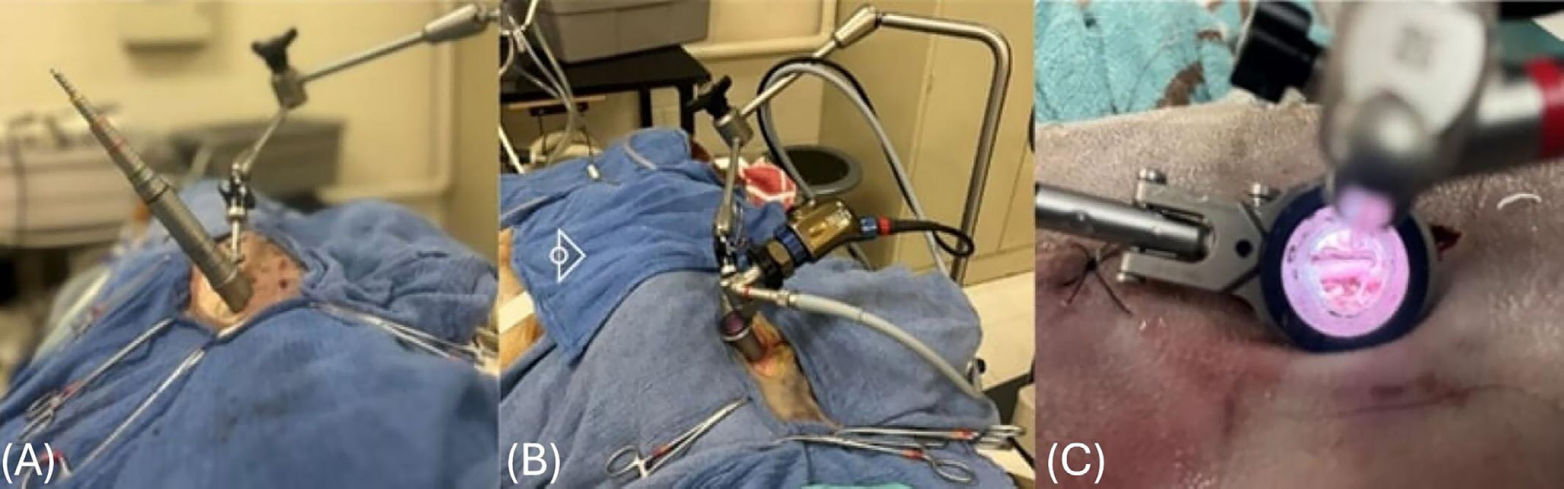
(A) Setup of the endoscopic tubular dilator system. (B) The endoscopic cannula, camera and light source are secured in place with an articulating locking arm secured to the table. (C) View of the mini‐hemilaminectomy and corpectomy through the endoscopic cannula.

All surgeries were performed by the same neurology resident (MW) after guidance and training from a board‐certified veterinary neurologist (LB) with experience in minimally invasive neurosurgery with this specific integrated endoscopic system. For each surgery, an approximately 2 cm incision was made at the level of the articular processes, incising through skin and subcutaneous tissue to expose the lumbodorsal fascia. The articular processes were located by palpation. A stab incision was made into the lumbodorsal fascia using a #11 blade, and a Kirschner wire (DePuy Synthes; West Chester, Pennsylvania) was inserted through this incision into the dorsolateral aspect of the articular processes. This was used as a guide wire for placement of the integrated endoscopic tubular dilator system (EasyGO! II Generation; Karl Storz Veterinary Endoscopy, Goleta, California). The smallest dilation sleeve was placed over the Kirschner wire to contact the articular processes, then subsequent dilation sleeves of increasing diameter were placed using a previously reported technique.^10^, ^11^ After a total of seven dilation sleeves were placed, a 23 mm cannula was inserted over the largest dilation sleeve, and the dilation sleeves were removed. The cannula was positioned approximately 30° laterally from the spinous process and secured in place with an articulating locking arm (Karl Storz Veterinary Endoscopy) clamped to the table. A 25° telescope was attached to the cannula, and a camera and light source were connected to the telescope (Figure 1). The remaining procedure was performed through the cannula with video endoscopy.

Once the cannula was centered on the articular processes, the entire system was manually redirected caudoventrally and centered over the accessory process and corresponding neurovascular bundle. If surrounding musculature elevated off the articular processes fell back into the field of view, the tubular dilators were carefully adjusted to re‐establish visualization of the surgical field. Once the endoscopic system was secured in place over the accessory process, remaining musculature and tendinous attachments to the accessory process and adjacent lamina were removed with sharp and blunt dissection.

**FIGURE 2:**
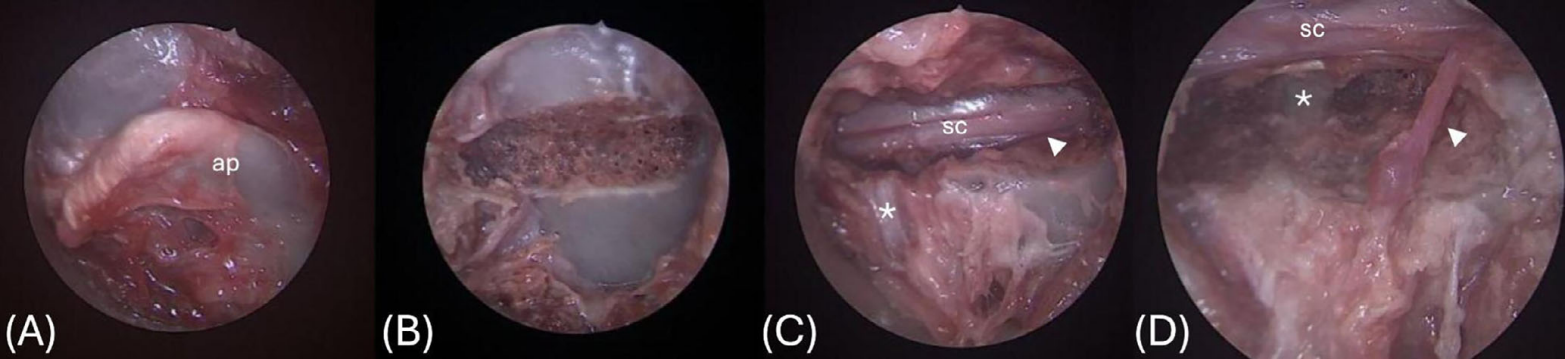
View of the mini‐hemilaminectomy and corpectomy as seen on the endoscopy video monitor. (A) The accessory process (ap) prior to drilling. (B) Drilling of the mini‐hemilaminectomy. (C) Completed mini‐hemilaminectomy with the visible spinal cord (sc), nerve root (white arrowhead), and intervertebral disc space (asterisk). (D) Completed corpectomy with the visible spinal cord (sc), nerve root (white arrowhead), and intervertebral disc space (asterisk).

A high‐speed electric drill and round burr (Stryker, Kalamazoo, Michigan) were used to first complete a mini‐hemilaminectomy extending as far cranially and caudally along the lamina as needed to visualize the spinal cord, nerve roots, intervertebral disc, and adjacent vertebral end plates. The cannula was gently repositioned cranially or caudally as needed to extend the drilling window. Following mini‐hemilaminectomy, a partial corpectomy was performed as described previously,^5^ with the surgeon using the preoperative CT measurements for guidance to achieve targeted dimensions. Drill burr size was used to approximate the length and height of the corpectomy. A depth gauge was periodically inserted into the corpectomy slot through the endoscopic cannula to estimate corpectomy depth. Saline solution and suction were used intermittently to clear debris and smoke away from the surgical site. Throughout the process, the nerve root exiting the intervertebral foramen was retracted cranially using a nerve retractor as needed. Figure 2 shows the magnified view throughout the procedure as visualized on the endoscopy video monitor.

**FIGURE 3:**
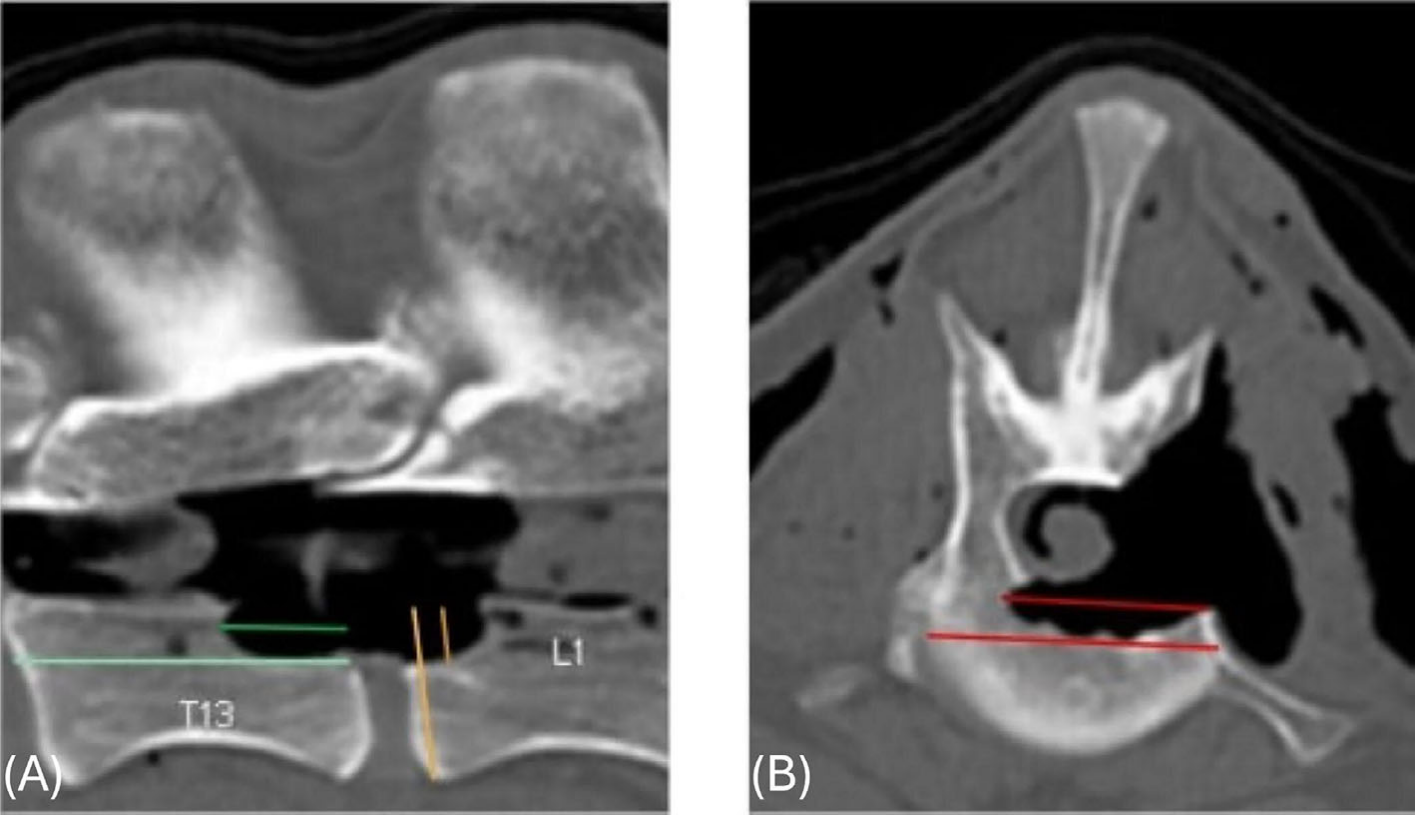
Postoperative (A) sagittal and (B) transverse computed tomography images showing measurement of the corpectomy length (short green), height (short orange), and depth (short red) as a proportion of normal vertebral dimensions (longer lines of each respective color).

A postoperative CT scan was performed on each cadaver using the same imaging protocol as for the preoperative scans (Figure 3). A veterinary radiology resident (KN) reviewed these images and measured corpectomy dimensions (height, width, and depth) for each surgical site at the same location as on the presurgical imaging. Surgical dimensions were recorded as a percentage of the normal vertebral dimensions. Due to small sample size and unequal numbers in each group, continuous data was described using median and interquartile range.

## 3 RESULTS

All eight cadavers were mixed breed dogs of unknown age, ranging from 17.2 to 28.0 kg. There were three males (2 intact, 1 castrated) and five females (reproductive status unknown). No CT evidence of intervertebral disc disease or other thoracolumbar vertebral pathology was noted for any cadaver.

Initially, a total of 48 surgeries were planned. Six surgeries were excluded due to the procedure being erroneously performed at the wrong location. Three surgeries were performed at L4–5 instead of L3–4, and one was performed at T10–11 instead of T11–12; these surgeries were excluded since they were not performed at a location of interest. In dog 2, two surgeries were erroneously done at L2–3 on opposite sides, and both sites were excluded. Notably, surgeries at T11–12 and T12–13 were unable to be performed in dogs 1, 3 or 4, due to difficulty with positioning the endoscopic cannula like due to inexperience. A total of 11 of the 12 excluded sites occurred in the first four cadavers. The 36 surgeries included for analysis were performed at T11–12 (*n* = 4), T12–13 (*n* = 5), T13–L1 (*n* = 8), L1–2 (*n* = 7), L2–3 (*n* = 7), and L3–4 (*n* = 5).

Median ± interquartile range (IQR) corpectomy dimensions were 23.07–± 16.47% of vertebral body length, 46.76– ± 21.7% of vertebral body height, and 74.44 ± 47.37% of vertebral canal diameter. Targeted dimensions were achieved at all intervertebral disc levels (Table 1).

**TABLE 1:** Corpectomy dimensions at each intervertebral disc space.

Intervertebral disc space	% Vertebral body length	% Vertebral body height	% Vertebral canal diameter
T11–12	32.56 ± 4.98	57.88 ± 18.62	83.94 ± 31.87
T12–13	27.17 ± 13.35	45.46 ± 33.14	66.91 ± 11.31
T13–L1	19.99 ± 13.81	49.34 ± 14.23	64.20 ± 63.79
L1–2	20.91 ± 13.71	39.89 ± 15.83	74.82 ± 34.62
L2–3	20.20 ± 13.61	49.25 ± 12.96	73.07 ± 20.92
L3–4	28.93 ± 16.84	38.53 ± 33.66	75.45 ± 25.25
All sites (*n* = 36)	23.07 ± 16.47	46.76 ± 21.7	74.44 ± 47.37

*Note*: Up to six surgeries were performed on each cadaver at T11–12 (*n* = 4), T12–13 (*n* = 5), T13–L1 (*n* = 8), L1–2 (*n* = 7), L2–3 (*n* = 7), and L3–4 (*n* = 5), alternating laterality between the left and right side.

^a^ All data are reported as median ± interquartile range.

A total of 13 surgeries were performed with the cadaver in sternal recumbency, and 23 were performed with the cadaver angled approximately 30° (*n* = 6) or 60° (*n* = 17) away from the surgeon. Subjectively, angled positioned altered the corpectomy drill trajectory in a way that facilitated more horizontal drilling across the vertebral body, resulting in a shorter corpectomy length and height compared to sternal recumbency (Table 2). Rhizotomy did not need to be performed in any procedure; subjectively, the nerve roots did not appear traumatized when visualized through the cannula at the end of each procedure. Rib head resection was not performed for any surgery as it was not required to obtain targeted surgical dimensions.

**TABLE 2:** Corpectomy dimensions with various surgical positioning.

Dimensions	Sternal recumbency	Angled away from surgeon
% vertebral body length	32.35 ± 4.42	17.29 ± 12.76
% vertebral body height	57.49 ± 21.23	39.89 ± 13.95
% vertebral canal diameter	38.13 ± 68.28	74.82 ± 19.88

*Note*: Cadavers were positioned either in sternal recumbency (*n* = 13) or angled 30°–60° away from the surgeon (*n* = 23).

^a^ Data are reported as median ± interquartile range.

## 4 DISCUSSION

Integrated endoscopic mini‐hemilaminectomy and corpectomy were successfully performed from the T11–12 through L3–4 intervertebral disc spaces. To the author's knowledge, this is the first study comparing minimally invasive mini‐hemilaminectomy and corpectomy at multiple locations in the canine vertebral column using an integrated endoscopic system.

The targeted surgical dimensions were achieved or exceeded at all intervertebral disc spaces from T11–12 through L3–4. Exceeding the target dimensions was not considered a failure since the primary goal was to assess if the conventionally accepted dimensions could be achieved with an endoscopic surgical system. These target dimensions are based on original publications of the corpectomy technique which primarily focused on spinal cord compression and not vertebral column stability.^5^ Although studies have demonstrated that corpectomy increased vertebral column range of motion in flexion/extension and lateral bending,^14^ case reports of clinically significant postoperative vertebral instability are lacking. Case reports of vertebral subluxation following cervical ventral slot suggest that a slot to vertebral body ratio greater than 0.5 may be associated with subluxation,^15^ but this has not been documented in thoracolumbar surgery. One study specifically evaluating corpectomy found that 27% of dogs had a corpectomy depth of >70% without clinical evidence of vertebral column instability, with one slot extending to 89% of vertebral body width.^6^ In another study, median slot depth ranged from 36% to 78.6% with no reported cases of clinically significant instability postoperatively.^7^ Further studies with multiple surgeons are needed to determine if there is a tendency to exceed planed dimensions with an endoscopic technique. In clinical practice, care should be taken to measure with a depth gauge throughout drilling to ensure the depth of the corpectomy slot is appropriate to adequately decompress the spinal cord based on preoperative imaging for that individual patient.

**FIGURE 4:**
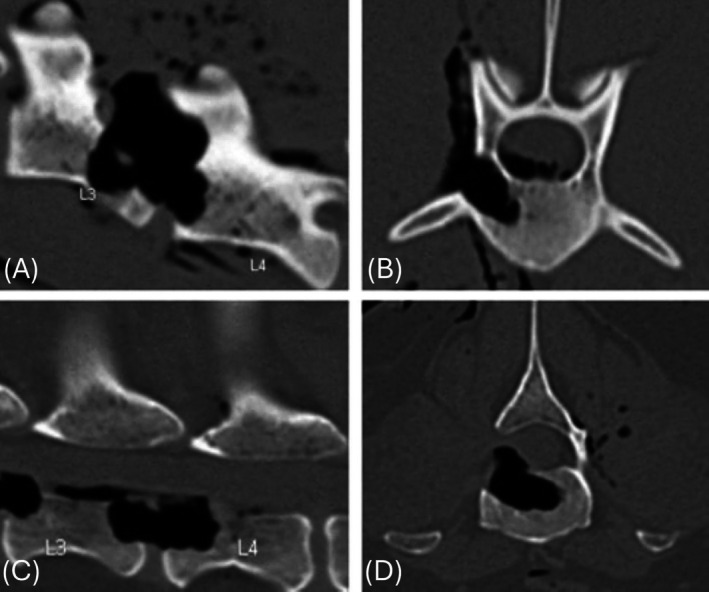
Postoperative sagittal and transverse computed tomography images showing position of the corpectomy with the cadaver positioned in (A, B) sternal recumbency and (C, D) when angled 60° away from the surgeon.

The primary outcome measure when performing a corpectomy is adequate spinal cord decompression, with corpectomy depth being the main factor that aids in spinal cord decompression.^6^, ^9^ In one retrospective study of 51 dogs, a deeper slot allowed for more complete decompression, but corpectomy length, height, and angle did not significantly influence the degree of decompression.^6^ In the present study, angled positioning subjectively made the approach to the vertebral body easier without impacting ability to reach target corpectomy depth. There was large intragroup variability given the wide range of measurements in the sternal recumbency group, as well as low study power due to small group numbers (type II error). Future larger studies are needed to identify a statistically significant difference in percentage of vertebral canal diameter achieved with angled versus sternal positioning. It is the authors' opinion that angled positioning overall provides easier access to the accessory process during the initial approach and facilitates easier drilling horizontally across the vertebral body (Figure 4).

Location in the thoracolumbar vertebral column did not affect the size of the corpectomy in this study. The chosen intervertebral disc spaces are the most common sites where a corpectomy is performed in clinical practice.^6^ Except for the German Shepherd dog,^16^, ^17^ intervertebral disc herniations in the cranial thoracic vertebrae are uncommon due to the presence of the intercapital ligament from T2–11,^9^, ^18^ and corpectomy is contraindicated caudal to L4 due to increased risk of iatrogenic damage to the nerve roots innervating the lumbosacral plexus. However, the biggest complication encountered was incorrect localization of the correct intervertebral disc space in six cases. This could likely be overcome with the use of intraoperative fluoroscopy, which was not available in the present study due to budgetary and equipment constraints. The authors also recognize that in the first four cadavers, positioning of the endoscopic cannula was more difficult in the thoracic vertebral column, preventing several surgeries from being completed. This was rectified in the last four cadavers as experience grew. Once the endoscopic system could successfully be placed, the actual surgical dimensions achieved were comparable to those of the lumbar sites.

Benefits of this integrated endoscopic technique include excellent visualization due to the magnification and illumination provided by the endoscopic camera and light source. This is especially beneficial for corpectomy, which carries several known potential complications including traumatic rhizotomy, need for rib head resection or luxation, and hemorrhage from the venous sinus.^5^, ^6^, ^7^ Using a 23 mm endoscopic tubular cannula, corpectomy could successfully be performed without the need to disrupt the rib head. Additionally, the nerve root could be visualized with excellent detail on the video monitor, and a blunt nerve hook could easily be inserted into the cannula for nerve root manipulation by an assistant during drilling of the corpectomy (Video Clip S1).

Another theorized benefit of this minimally invasive approach is preservation of normal epaxial muscular attachments which may further reduce postoperative instability. The intervertebral disc, one of the most important stabilizers of the canine vertebral column,^19^, ^20^ is intentionally disrupted during TLCC. Previous studies have shown that TLCC significantly increases the range of motion of the vertebral body unit.^10.14^ Epaxial muscles also contribute to stability and force production in the thoracolumbar vertebrae.^21^ Preservation of surrounding musculature therefore may reduce instability following TLCC, although biomechanical studies of the vertebral column following traditional versus minimally invasive TLCC would be required to confirm if this is clinically significant.

Because the Kirschner wire could inadvertently penetrate the intervertebral foramen and corresponding neurovascular bundle, the endoscopic cannula was first positioned over the articular process similar to the approach for integrated endoscopic hemilaminectomy.^22^, ^23^ The entire system was then redirected ventrally to be centered over the accessory process; the endoscopic camera is already in place at this point which allows for visualization of the neurovascular bundle while repositioning. In clinical practice it is possible that fluoroscopy may allow for more direct placement of the tubular dilator system over the accessory process. Another study using the same integrated endoscopic system for mini‐hemilaminectomy without corpectomy in 11 clinical patients used a more direct approach to the intervertebral foramina with fluoroscopic guidance, without documented evidence of significant trauma to the neurovascular bundle.^24^ In the present study, repositioning the system from the articular process to the accessory process did not appear to hinder the overall outcome. Using the same initial approach as for endoscopic hemilaminectomy may also simplify endoscopic neurosurgical training by allowing one similar approach to be applied to multiple procedures.

Because the study used cadavers, venous sinus hemorrhage could not be replicated. However, the venous sinus was easily visualized due to the magnification provided by the endoscopic camera. In studies of clinical patients undergoing integrated endoscopic hemilaminectomy or mini‐hemilaminectomy without corpectomy using this same endoscopic surgical system, significant hemorrhage and subsequent conversion to an open approach was a complication in 0%–9% of patients.^23^, ^24^ Future studies applying this technique to clinical patients will be needed to evaluate if similar outcomes are seen with integrated endoscopic corpectomy.

The main limitation of this study was the small sample size, and studies with larger numbers are required to confirm the findings in this pilot study. Another limitation was the fact that all surgeries were performed on cadavers without evidence of significant spinal cord compression on CT. Although surgical dimensions in this study mimicked the typical target dimensions used in live patients, removal of compressive intervertebral disc material was not replicated in this model. Additionally, multiple surgeries were performed on each cadaver, which does not exactly replicate the typical clinical scenario. However, because laterality was alternated, no two surgeries were directly adjacent to each other, so the effect of this is expected to be minimal.

All cadavers in this study were medium to large breeds, since this is most representative of the patient signalment most likely to undergo corpectomy in clinical practice. However, corpectomy has also been used for decompression of herniated discs in small‐breed and/or chondrodystrophic patients such as the Dachshund.^6^ A recent study of integrated endoscopic hemilaminectomy in clinical patients utilized a 19 mm cannula in dogs as small as 3.4 kg with good outcomes.^23^ It is suspected that integrated endoscopic TLCC can similarly be applied to small breeds and/or chondrodystrophic patients with a smaller cannula size, but further studies are needed to confirm this. Finally, a single surgeon performed all surgeries in this study for standardization. A learning curve certainly exists even for experienced surgeons when first using the endoscopic video system, and results are likely to be variable with individual skill level and comfort with drilling with an indirect view of the spinal canal through the endoscopy video monitor; studies with multiple surgeons of various experience levels are needed. Length of surgical procedure was not recorded in this study, but it is the authors' experience that endoscopic spinal surgery takes longer than a conventional open approach.

In conclusion, mini‐hemilaminectomy and corpectomy are feasible throughout the canine thoracolumbar vertebral column using an integrated endoscopic surgical system. Subjectively, surgery of the thoracic vertebral column involved a steeper learning curve, but targeted dimensions could be achieved. Angled positioning may affect some surgical dimensions but is considered unlikely to significantly affect surgical outcome, as adequate corpectomy depth could be achieved in all surgeries. Future studies in clinical patients with intervertebral disc disease are required to assess the utility of this approach in achieving successful patient outcomes.

## AUTHOR CONTRIBUTIONS

Wolfe MM, VMD: Performed all surgeries, compiled and interpreted data, performed statistical analysis, and drafted and revised the manuscript. Bartner LR, DVM, MS, DACVIM (Neurology): Contributed to study design, oversaw surgical procedures, and provided manuscript revision. Monnet E, DVM, PhD, DACVS, ECVS: Contributed to the study design, provided guidance regarding the use of endoscopic surgical equipment, and provided manuscript revision. Neal K. DVM, MS, DACVR: Performed radiographic measurements and manuscript revision. All authors critically reviewed the manuscript and endorsed the final version. All authors are aware of their contributions and have confidence in the integrity of the manuscript.

## Supporting information


**Video Clip S1.** Videoendoscopy demonstrating drilling of the corpectomy in cadaver Dog 8. The cranial end plate is drilled while the nerve root is retracted by an assistant.
